# Supplementary material for: Structural basis for aminoacylation of cellular modified tRNALys3 by human lysyl-tRNA synthetase
Source: Nucleic Acids Res. 2025 Feb 27;53(5):gkaf114. doi: 10.1093/nar/gkaf114 (PMC11878792; doi:10.1093/nar/gkaf114)
Supplement: gkaf114_Supplemental_Files [file gkaf114_supplemental_files.zip › Supplementary Data_Jan 31.pdf]

Figure S1

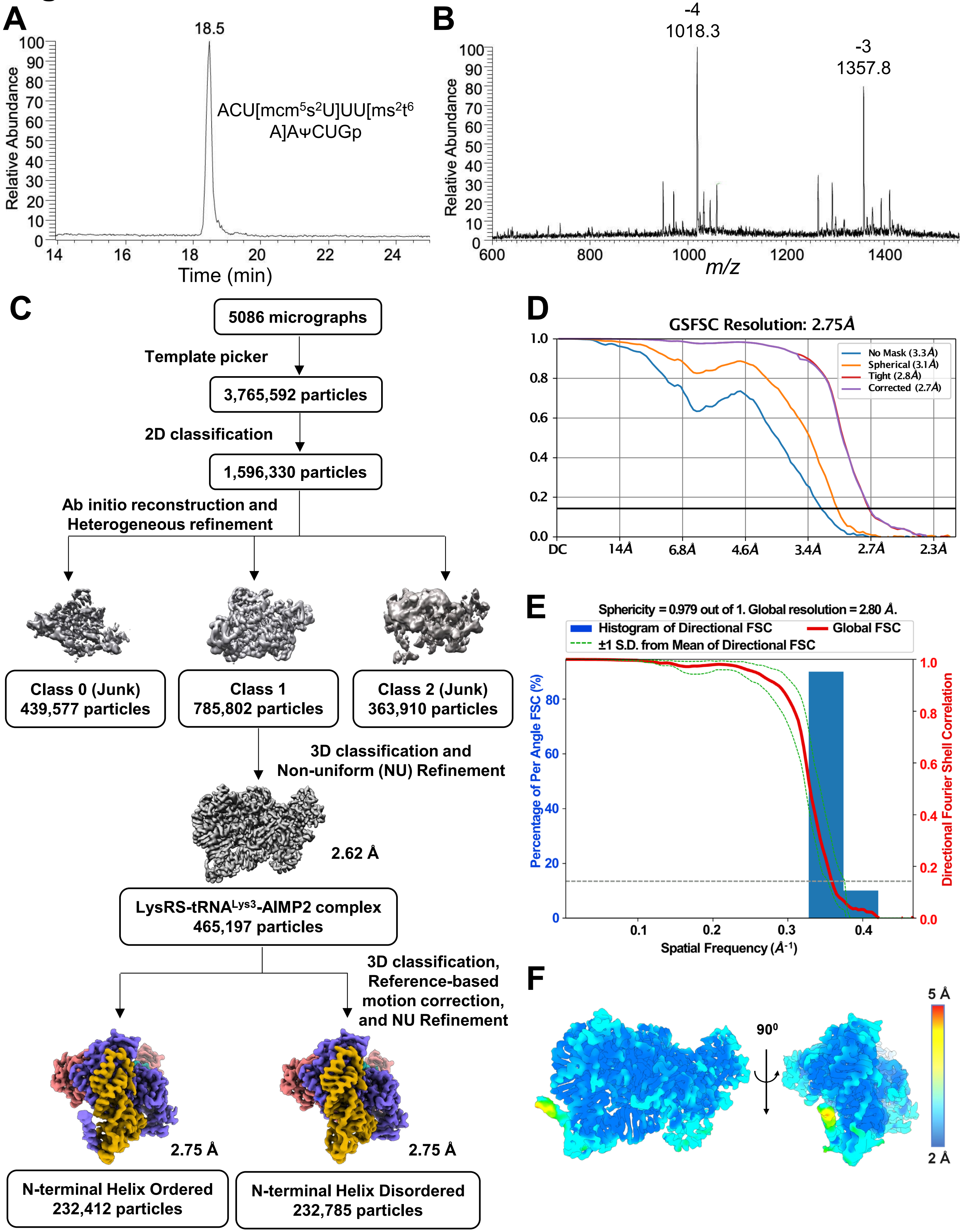
A
18.5
ACU[mcm5s2U]UU[ms2t6 A]AΨCUGp
Relative Abundance
Time (min)
B
-4
1018.3
-3
1357.8
Relative Abundance
m/z
C
5086 micrographs
Template picker
3,765,592 particles
2D classification
1,596,330 particles
Ab initio reconstruction and Heterogeneous refinement
Class 0 (Junk) 439,577 particles
Class 1 785,802 particles
Class 2 (Junk) 363,910 particles
3D classification and Non-uniform (NU) Refinement
2.62 Å
LysRS-tRNALys3-AIMP2 complex 465,197 particles
3D classification, Reference-based motion correction, and NU Refinement
2.75 Å
2.75 Å
N-terminal Helix Ordered 232,412 particles
N-terminal Helix Disordered 232,785 particles
D
GSFSC Resolution: 2.75Å
No Mask (3.3Å)
Spherical (3.1Å)
Tight (2.8Å)
Corrected (2.7Å)
DC
14Å
6.8Å
4.6Å
3.4Å
2.7Å
2.3Å
E
Sphericity = 0.979 out of 1. Global resolution = 2.80 Å.
Histogram of Directional FSC
Global FSC
±1 S.D. from Mean of Directional FSC
Percentage of Per Angle FSC (%)
Directional Fourier Shell Correlation
Spatial Frequency (Å-1)
F
90⁰
5 Å
2 Å

Figure S1. (A) Representative extracted ion chromatogram (EIC) of the summation of ions detected for the RNase T1 digestion product ‘ACU[mcm^{5}s^{2}U]UU[ms^{2}t^{6}A]AYCUGp’ from cellular modified tRNA^{Lys3} eluting at 18.48 min. **(B)** Mass spectrum of the RNase T1 digestion product ‘ACU[mcm^{5}s^{2}U]UU[ms^{2}t^{6}A]AYCUGp’ from tRNA^{Lys3}. The calculated molecular weight for this digestion product is 4077.6 and the -3 and -4 charge states are labeled in this spectrum. **(C)** Overall data processing workflow for the LysRS-cellular modified tRNA^{Lys3}-AIMP2 cryo-EM reconstruction. **(D)** The gold-standard Fourier Shell Correlation (GS-FSC) curve for the LysRS-cellular modified tRNA^{Lys3}-AIMP2 reconstruction is shown. **(E)** 3D-FSC plot for the LysRS-cellular modified tRNA^{Lys3}-AIMP2 reconstruction showing the directional resolution anisotropy. An overall sphericity of 0.98 shows that the reported global resolution of 2.8 Å is uniform in all three dimensions and the sample does not show major preferred orientations. **(F)** The local resolution for the final cryo-EM reconstruction of the LysRS-cellular modified tRNA^{Lys3}-AIMP2 complex is shown in a color-coded format.

Figure S2

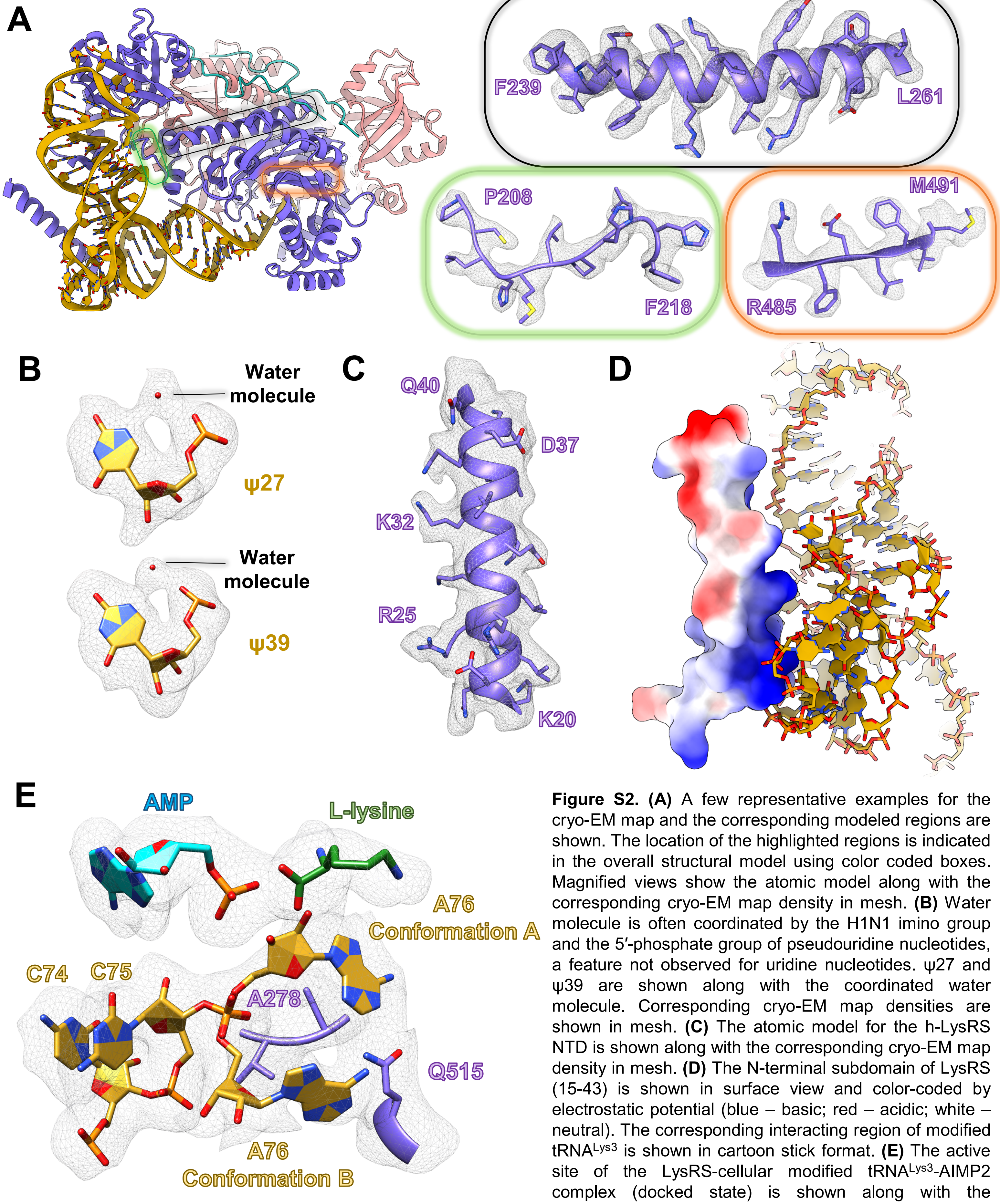

Figure S2. (A) A few representative examples for the cryo-EM map and the corresponding modeled regions are shown. The location of the highlighted regions is indicated in the overall structural model using color coded boxes. Magnified views show the atomic model along with the corresponding cryo-EM map density in mesh. **(B)** Water molecule is often coordinated by the H1N1 imino group and the 5′-phosphate group of pseudouridine nucleotides, a feature not observed for uridine nucleotides. ψ27 and ψ39 are shown along with the coordinated water molecule. Corresponding cryo-EM map densities are shown in mesh. **(C)** The atomic model for the h-LysRS NTD is shown along with the corresponding cryo-EM map density in mesh. **(D)** The N-terminal subdomain of LysRS (15-43) is shown in surface view and color-coded by electrostatic potential (blue – basic; red – acidic; white – neutral). The corresponding interacting region of modified $tRNA^{Lys3}$ is shown in cartoon stick format. **(E)** The active site of the LysRS-cellular modified $tRNA^{Lys3}$-AIMP2 complex (docked state) is shown along with the corresponding cryo-EM map density to highlight the two alternate conformations of the A76 nucleotide of $tRNA^{Lys3}$.

Figure S3

A

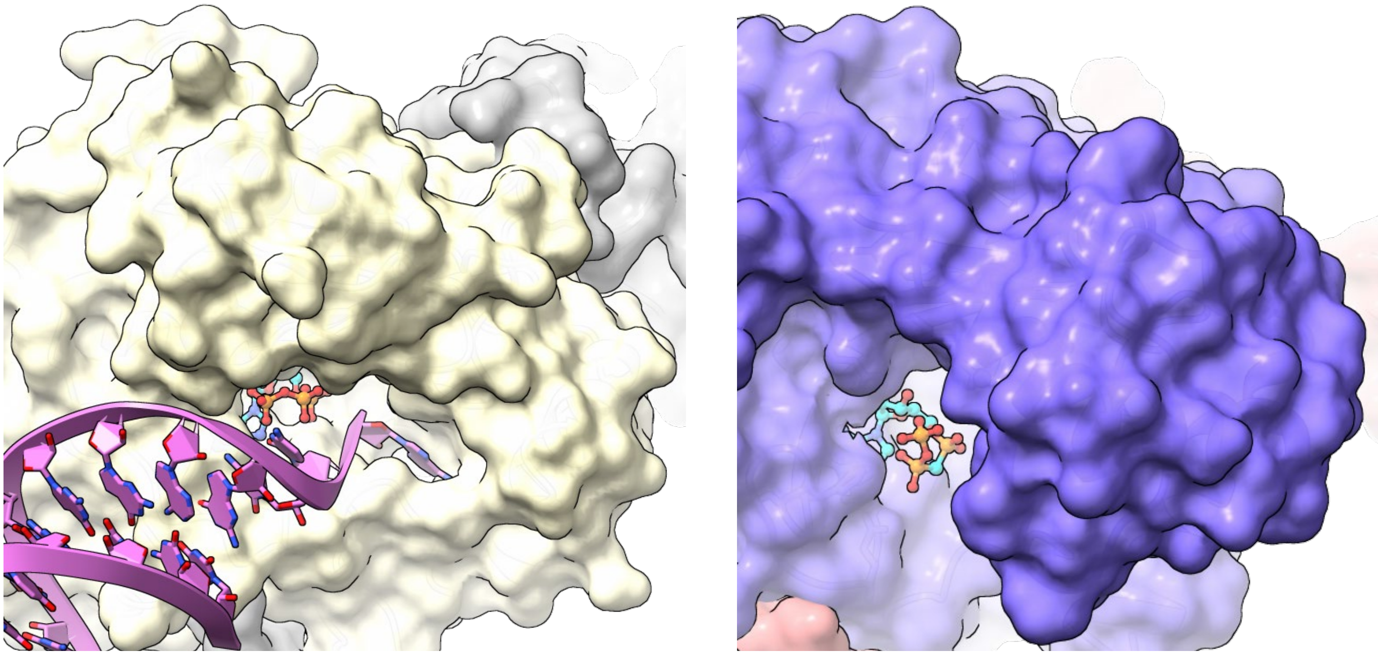

B

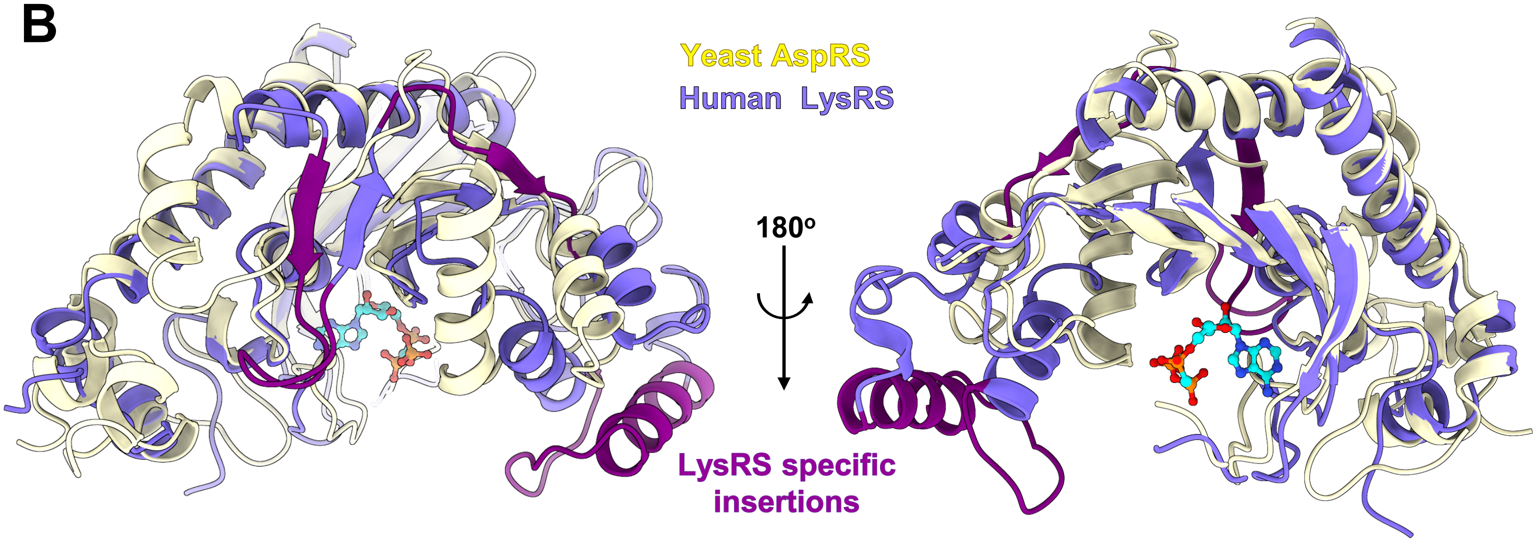

C

```
sp|Q15046|SYK_HUMAN      IETPMMNIIPGGAVAKPFITYHNELDMNLYMRIAPELYHKMLVVGGIDRVYEIGRQFRNE  325
sp|P04802|SYDC_YEAST     VHTPKLLGAPSEGGSSVFE--VTYFKGKAYLAQSPQFNKQQLIVADFERVYEIGPVFRAE  327
                         :.** :   *. . :. *    . :. : *:  :*:: :: *:*..::******  ** *

sp|Q15046|SYK_HUMAN      GIDL-THNPEFTTCEFYMAYAD-YHDLMEITEKMVSGMVKHITGSYKVTYHPDGPEGQAY  383
sp|P04802|SYDC_YEAST     NSNTHRHMTEFTGLDMEMAFEEHYHEVLDTLSELFVFIFSELPKRF-----------AH  375
                         . :   *  ***  :: **: : **::::  .::.  :...:   :            *:

sp|Q15046|SYK_HUMAN      DVDFTPPFRRINMVEELEKALGMKLPETNLFETEETRKILDDICVAKAVECPPPRTTARL  443
sp|P04802|SYDC_YEAST     EIEL---------VRKQYPVEEFKLPKDGKMVRLTY---------KEGI------EMLRA  411
                         ::::         *.:   .  :***: . :               :.:         *
```

Figure S3. (A) A side-by-side view of the active sites of related class-II AARS, yeast AspRS (left panel, yellow) and human LysRS (right panel, purple), in the ATP bound state is shown. The entrance for the LysRS active site is considerably narrow compared to yeast AspRS, and unlike yeast AspRS, the simultaneous binding of ATP and the tRNA 3'-CCA end is incompatible in the LysRS active site. **(B)** The structures of the yeast AspRS-tRNA^{Asp} complex (yellow) and human LysRS-tRNA^{Lys3} complex (purple) were aligned and the aminoacylation domains (AAD) for both are shown in cartoon format. Despite high degrees of conservation, LysRS AAD has insertions (highlighted in deep purple) that lead to a narrow entrance to its active site, in contrast to AspRS. **(C)** Sequence alignment between yeast AspRS (Uniprot: P04802) and human LysRS (Uniprot: Q15046) was carried out using Clustal Omega and is shown to highlight the LysRS-specific insertions (highlighted in purple box).

Figure S4

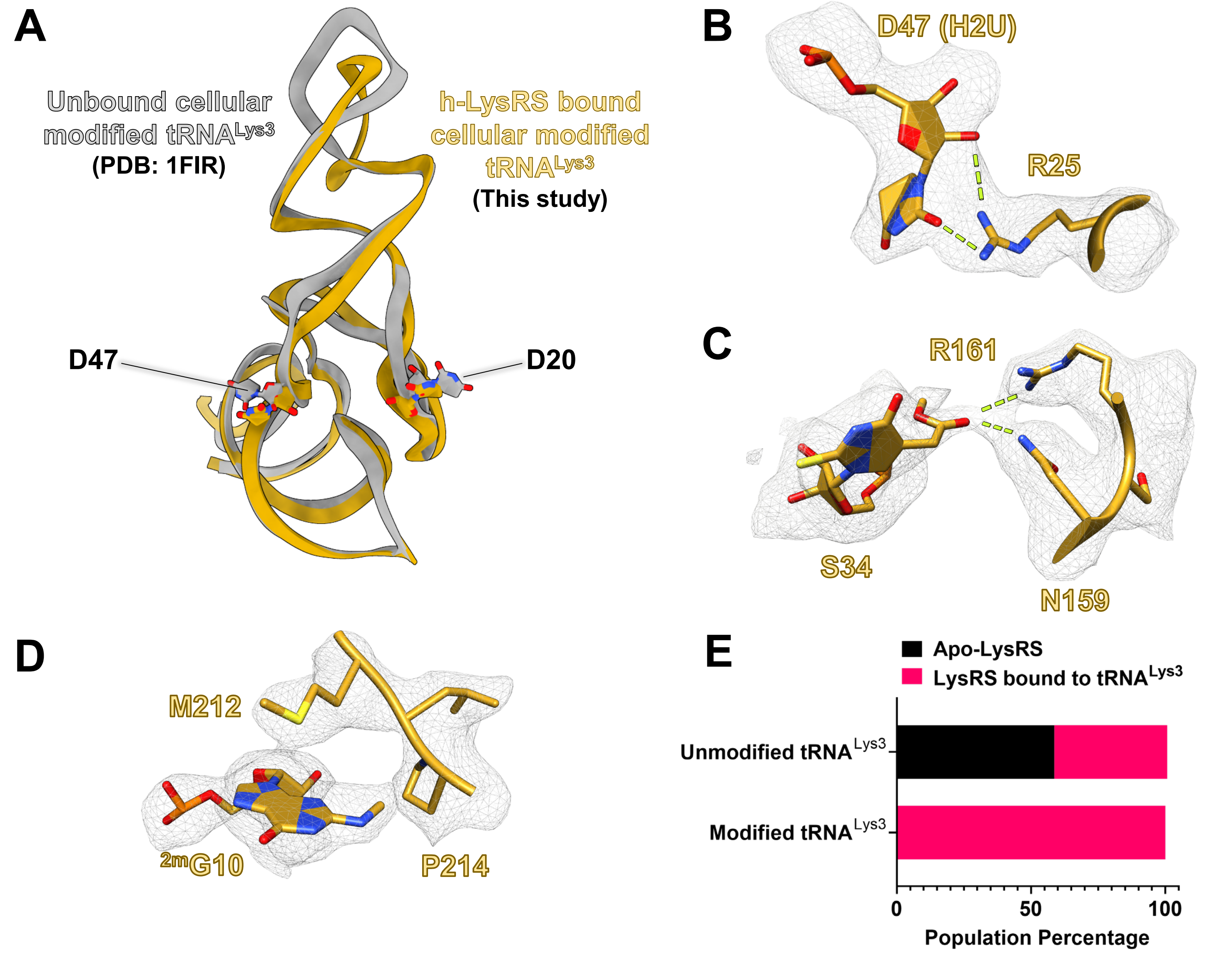

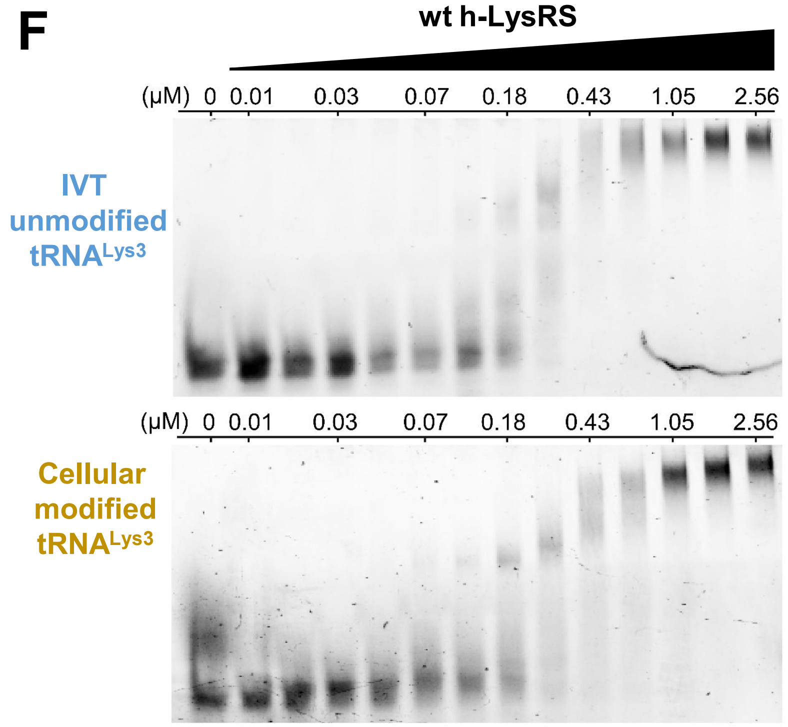

Figure S4. (A) Cellular modified tRNA^{Lys3} from the h-LysRS bound complex (gold, this study) was aligned to its unbound form (gray, PDB: 1FIR). The modified nucleotides D20 and D47 are highlighted and assume an unstacked conformation in both the unbound and h-LysRS bound state. **(B-D)** LysRS specifically recognizes the tRNA^{Lys3} modifications and a few representative examples are shown along with the corresponding cryo-EM map density. The three examples shown here are D47 (5,6 dihydrouracil) (B), S34 (5-methoxycarbonylmethyl-2-thiouridine) (C), and ^{m2}G10 (2-methyl guanosine) (D). **(E)** Population statistics of h-LysRS from the cryo-EM dataset for cellular modified tRNA^{Lys3} (+AIMP2) and IVT unmodified tRNA^{Lys3} (+AIMP2) are shown in bar chart form. **(F)** A representative EMSA gel showing h-LysRS titration against IVT unmodified and cellular modified tRNA^{Lys3}.

Figure S5

A

```
sp|O43776|SYNC_HUMAN      MVLAELYVSDREGSDATGDGTKEKPFKTGLKALMTVGKEPFPTIYVDSQKENERWNVISK
sp|Q15046|SYK_HUMAN       -------MAAVQAAEVKVDGSEPKLSKNELKRRLKAEKKV-------AEKEAKQKELSEK
sp|P14868|SYDC_HUMAN      ----------------MPSASASRKSQEKPREIMDAAEDY----------AKERYGISSM
                                            ..:  .  :   .  : . :.             :.  : .

sp|O43776|SYNC_HUMAN      SQLKNIKKMWHREQMKSESREKKEAEDSLRREKNLEEAKKITIKNDPSLPEPKC--
sp|Q15046|SYK_HUMAN       QLSQATAAATNHTTDNGVGPEEESVDPNQYYKIRSQAIHQLKVNGEDPYPHKFHVD
sp|P14868|SYDC_HUMAN      IQSQEK-------------------------------------------PDRVL--
                             :                                             *
```

B

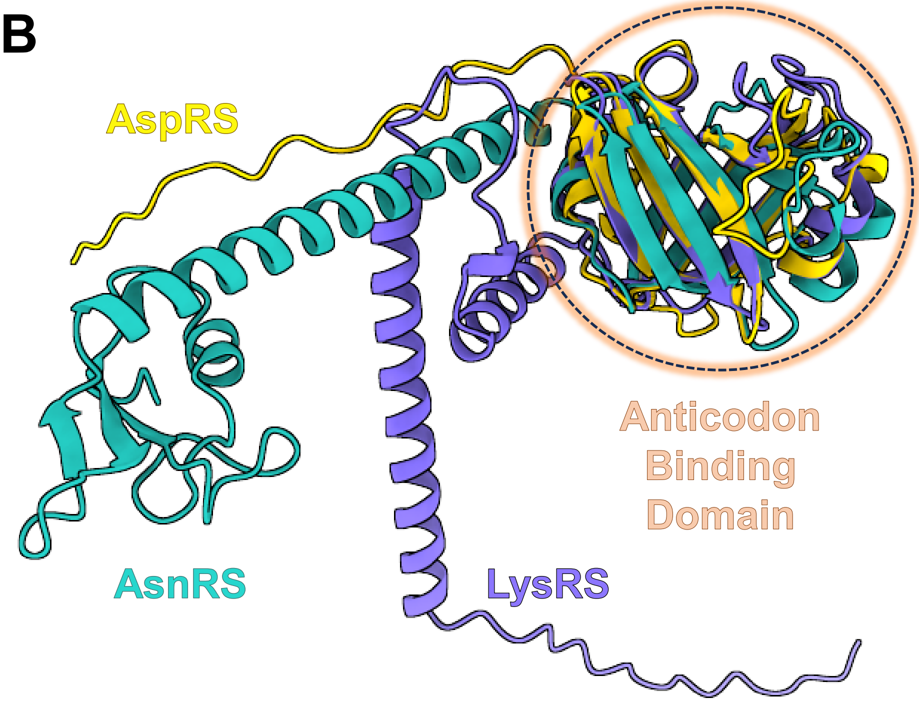

Figure S5. (A) The N-terminal extension domains of h-LysRS (Q15046, 1-102), h-AspRS (P14868, 1-45), and h-AsnRS (O43776, 1-114) were aligned using MUSCLE multiple sequence alignment algorithm. h-LysRS NTD residues that participate in tRNA^{Lys3} binding are highlighted by golden dashes. **(B)** The AlphaFold models for the N-terminal extension domains and anticodon-binding domains of h-LysRS (purple), h-AspRS (yellow), and h-AsnRS (sea green) were aligned in ChimeraX and are depicted in ribbon cartoon. The anticodon-binding domains of the three class-IIb AARS align well and are highlighted (orange circle).

Figure S6

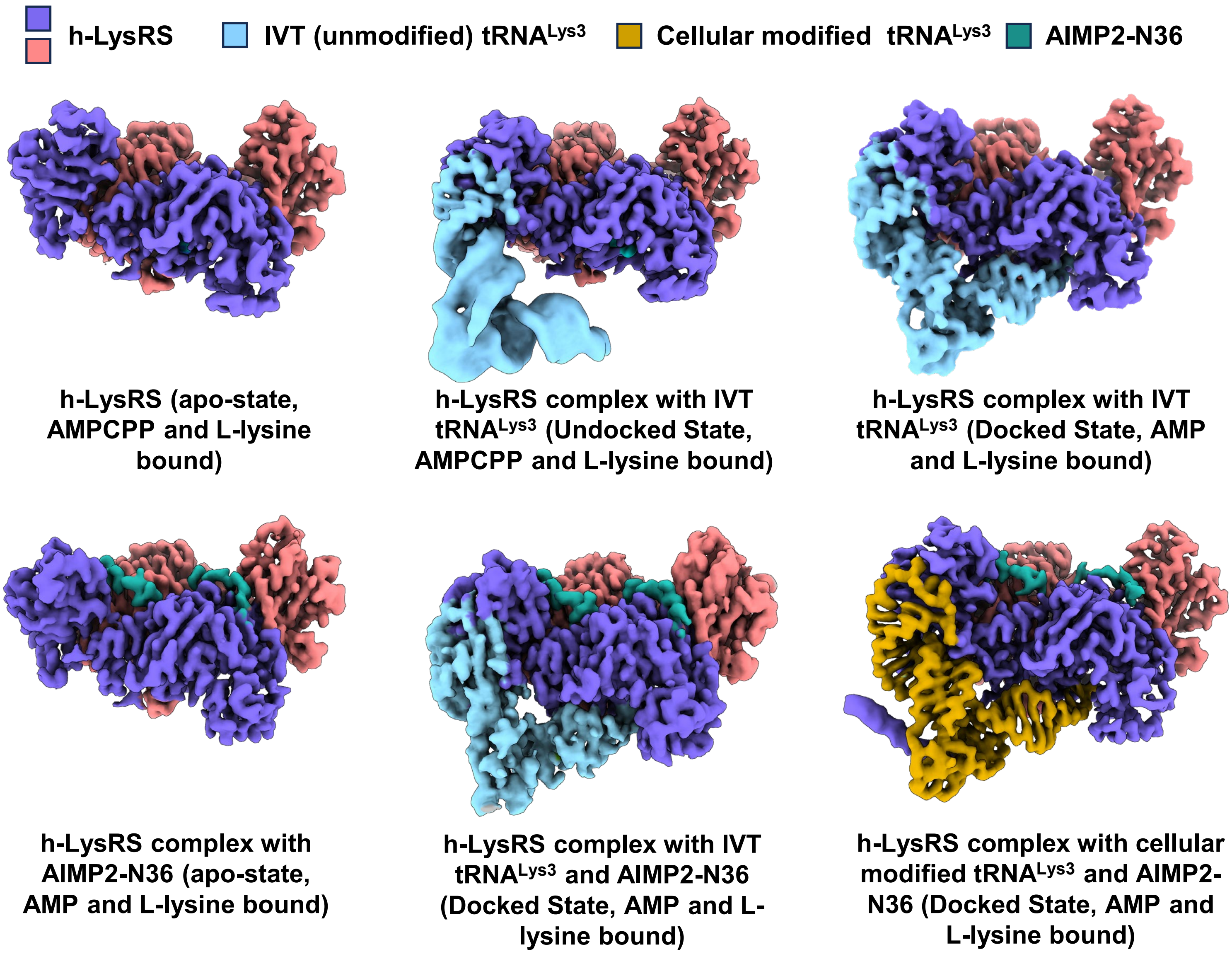

Figure S6. A summary of the distinct conformational states of h-LysRS complexes resolved in the cryo-EM datasets collected for this study.

Table S1

Mass Spectrometry Analysis for Modified Cellular $tRNA^{Lys3}$

Digestion Product	Theoretical *m/z*	Experimental *m/z*
**CCCGp**	(-1) 1277.8<br>(-2) 638.38	(-1) 1277.2<br>(-2) 638.25
**AUA[m^{2}G]p**	(-1) 1340.8<br>(-2) 669.91	(-1) 1340.1<br>(-2) 669.83
**CUCAGp**	(-1) 1608.0<br>(-2) 803.48	(-1) 1607.0<br>(-2) 803.25
**DCGp**	(-1) 975.59	(-1) 975.17
**DAGp**	(-1) 999.61	(-1) 999.17
**CAΨCAGp**	(-2) 968.08<br>(-3) 645.05	(-2) 967.67<br>(-3) 645.00
**ACU[mcm^{5}s^{2}U]UU[ms^{2}t^{6}A]AΨCUGp**	(-3) 1358.2<br>(-4) 1018.4	(-3) 1357.8<br>(-4) 1018.3
**[m^{7}G]UC[m^{5}C]AGp**	(-2) 990.11	(-2) 990.67
**[m^{5}Um]ΨCA[m^{1}A]Gp**	(-2) 989.62<br>(-3) 659.41	(-2) 989.25<br>(-3) 659.33
**UCCCUGp**	(-2) 944.55<br>(-3) 629.36	(-2) 944.33<br>(-3) 629.33
**UUCGp**	(-2) 639.36	(-2) 639.33
**CCA-OH**	(-1) 876.60	(-1) 876.17

Table S1. All RNase T1 digestion products from cellular modified $tRNA^{Lys3}$ detected by LC-MS. The sequences were confirmed by MS/MS data, except for the anticodon and variable loop region, which were analyzed manually. Both theoretical *m/z* values and those experimentally determined are listed.

Table S2

Cryo-EM Data Collection and Processing

	h-LysRS bound to modified $tRNA^{Lys3}$ and AIMP2	h-LysRS bound to IVT $tRNA^{Lys3}$	h-LysRS bound to IVT $tRNA^{Lys3}$	h-LysRS bound to IVT $tRNA^{Lys3}$ and AIMP2
**Ligands**	AMP and L-lysine	AMPCPP and L-lysine	AMP and L-lysine	AMP and L-lysine
**Number of micrographs**	4746	4643	5320	4045
**Magnification**	81,000x	81,000x	81,000x	81,000x
**Voltage (kV)**	300 kV	300 kV	300 kV	300 kV
**Electron exposure (e^{-}/Å^2)**	50 e^{-}/Å^2	50 e^{-}/Å^2	50 e^{-}/Å^2	50 e^{-}/Å^2
**Defocus range (µm)**	- 1.0 to -2.0	- 1.0 to -2.0	- 1.0 to -2.0	- 1.0 to -2.0
**Pixel size (Å)**	1.07 Å physical pixel size	1.07 Å physical pixel size	1.07 Å physical pixel size	1.07 Å physical pixel size
**Microscope**	Titan Krios	Titan Krios	Titan Krios	Titan Krios
**Detector**	Gatan K3	Gatan K3	Gatan K3	Gatan K3
**Data Collection Software**	EPU	EPU	EPU	EPU
**Data Processing Software**	cryoSPARC v4	cryoSPARC v4	cryoSPARC v4	cryoSPARC v4
**Symmetry imposed**	C1	C1	C1	C1
**Initial particle images (no.)**	785,802	881,907	1,211,344	601,206
**Final particle images (no.)**	232,412	430,648	302,069 (Docked state) 267,590 (Undocked state)	165,298
**FSC threshold and Map resolution (Å)**	0.143 FSC cutoff 2.7 Å (Docked state)	0.143 FSC cutoff 2.8 Å (Undocked state)	0.143 FSC cutoff 3 Å (Docked state) 3.1 Å (Undocked state)	0.143 FSC cutoff 4 Å (Docked state)
**Resolution range (Å)**	2.1 Å – 6.5 Å	2.3 Å – 10 Å	2.3 Å – 10 Å	2.4 Å – 11 Å

Table S2. Details of cryo-EM data collection and data processing for the four different datasets are presented in a tabular format.

Table S3

Model Building Statistics and Accession Codes

	LysRS bound to cellular modified $tRNA^{Lys3}$ and AIMP2 (Docked State)	LysRS bound to IVT $tRNA^{Lys3}$ (Undocked State)	LysRS bound to IVT $tRNA^{Lys3}$ (Docked State)	LysRS bound to IVT $tRNA^{Lys3}$ (Undocked State)
**Ligands**	AMP and L-lysine	AMPCPP and L-lysine	AMP and L-lysine	AMP and L-lysine
**Deposition ID**	PDB: 9DPL<br>EMD-47106	PDB: 9DPB<br>EMD-47101	PDB: 9DPA<br>EMD-47100	PDB: 9DOW<br>EMD-47094
**Map sharpening B factor ($Å^2$)**	- 55	- 76	- 45	- 33
**Initial Model Used**	PDB: 6ILD, 1FIR	PDB: 6ILD, 1FIR	PDB: 6ILD, 1FIR	PDB: 6ILD, 1FIR
**Model composition**				
**Non-hydrogen atoms**	10555	9836	9878	9816
**Protein residues**	1092	1013	1013	1013
**Nucleotides**	76	73	76	73
**Metal Ions**	4	5	1	1
**R.m.s. deviations**				
**Bond lengths (Å)**	0.01	0.006	0.007	0.005
**Bond angles (°)**	1.327	1.002	1.032	0.955
**Validation**				
**MolProbity score**	1.68	1.85	1.93	1.89
**Clashscore**	9.12	9.93	10.63	10.10
**Ramachandran plot**				
**Favored (%)**	96.94	96.72	96.43	96.62
**Allowed (%)**	3.06	3.28	3.57	3.38
**Disallowed (%)**	0.00	0.00	0.00	0.00

Table S3. Model building, data deposition and validation statistics.

Table S4

Sequence Information and Coverage for the Structural Models

Name	Alternative Name	Chain ID	Length (residues)	Modeled residues	UniProt ID (proteins)
**h-LysRS bound to cellular modified tRNA^{Lys3} and AIMP2-N36 (AMP and L-lysine)<br>PDB: 9DPL**					
**h-LysRS**	**KARS**	A	597	14-43, 69-579	Q15046
**h-LysRS**	**KARS**	B	597	71-577	Q15046
**tRNA^{Lys3}**	**-**	C	76	76	HG983931.1 (GenBank)
**AIMP2-N36**	**-**	D and E	36	1-21	Q13155
**h-LysRS bound to IVT tRNA^{Lys3} and AIMP2 (AMPCPP and L-lysine) [Undocked State]<br>PDB: 9DPB**					
**h-LysRS**	**KARS**	A and B	597	72-577 (A) and 72-576 (B)	Q15046
**tRNA^{Lys3}**	**-**	C	76	73	HG983931.1 (GenBank)
**h-LysRS bound to IVT tRNA^{Lys3} and AIMP2 (AMP and L-lysine) [Docked State]<br>PDB: 9DPA**					
**h-LysRS**	**KARS**	A and B	597	72-576 (A, B)	Q15046
**tRNA^{Lys3}**	**-**	C	76	76	HG983931.1 (GenBank)
**h-LysRS bound to IVT tRNA^{Lys3} and AIMP2 (AMP and L-lysine) [Undocked State]<br>PDB: 9DOW**					
**h-LysRS**	**KARS**	A and B	597	72-576 (A) and 72-577 (B)	Q15046
**tRNA^{Lys3}**	**-**	C	76	73	HG983931.1 (GenBank)

Table S4. Details of the four structural models including the Chain IDs, total length, modeled residues, and Uniprot/Genbank IDs are listed.

Table S5

Conformational States and Population Statistics

Dataset	Ligand	Apo-LysRS	h-LysRS-tRNA^{Lys3} complex	
			Undocked State	Docked State
**h-LysRS with cellular modified tRNA^{Lys3} and AIMP2**	AMP and L-lysine	0	0	465,197 (100%)
**h-LysRS with IVT tRNA^{Lys3}**	AMPCPP and L-lysine	458,550 (52%)	430,648 (48%)	0
**h-LysRS with IVT tRNA^{Lys3}**	AMP and L-lysine	268,855 (32%)	267,590 (32%)	302,069 (36%)
**h-LysRS with IVT tRNA^{Lys3} and AIMP2**	AMP and L-lysine	235,233 (59%)	0	165,298 (41%)

Table S5. Details of the distinct conformational states resolved in the four cryo-EM datasets. The final number of particles for each of the indicated conformational states are tabulated. The relative percentage of the particle stacks belonging to each conformational state in a dataset is presented in parenthesis.

Supplementary Movie S1. h-LysRS remodels the anticodon loop of $tRNA^{Lys3}$. This video highlights the remodeling of modified $tRNA^{Lys3}$ upon binding by h-LysRS. All 5 nucleotides in the anticodon loop are unstacked and involved in binding h-LysRS. The video was made using UCSF ChimeraX.

Supplementary Movie S2. $tRNA^{Lys3}$ modifications mediate recognition by h-LysRS. This video highlights the multiple identity elements used by h-LysRS for binding cellular modified $tRNA^{Lys3}$ and the integral role played by $tRNA^{Lys3}$ modifications. The video was made using UCSF ChimeraX.

Supplementary Movie S3. Conformational Landscape of $tRNA^{Lys3}$ aminoacylation. This video highlights the 3'-CCA undocked state and 3'-CCA docked state conformations of h-LysRS-$tRNA^{Lys3}$ complex (unmodified $tRNA^{Lys3}$) and how ATP governs the conformational dynamics of 3'-CCA end docking in the active site. The ordering of the two catalytic steps in $tRNA^{Lys}$ aminoacylation by h-LysRS is markedly different to previously reported aminoacylation pathway of yeast AspRS, a related class-II aminoacyl-tRNA synthetase. The video was made using UCSF ChimeraX.
